# Accessing local electron-beam induced temperature changes during *in situ* liquid-phase transmission electron microscopy†

**DOI:** 10.1039/d0na01027h

**Published:** 2021-02-19

**Authors:** Birk Fritsch, Andreas Hutzler, Mingjian Wu, Saba Khadivianazar, Lilian Vogl, Michael P. M. Jank, Martin März, Erdmann Spiecker

**Affiliations:** Electron Devices (LEB), Department of Electrical, Electronic and Communication Engineering, Friedrich-Alexander University Erlangen-Nürnberg (FAU) Cauerstraße 6 91058 Erlangen Germany andreas.hutzler@fau.de; Institute of Micro- and Nanostructure Research (IMN) & Center for Nanoanalysis and Electron Microscopy (CENEM), Interdisciplinary Center for Nanostructured Films (IZNF), Department of Materials Science and Engineering, Friedrich-Alexander University Erlangen-Nürnberg (FAU) Cauerstraße 3 91058 Erlangen Germany; Fraunhofer Institute for Integrated Systems and Device Technology IISB Schottkystraße 10 91058 Erlangen Germany

## Abstract

A significant electron-beam induced heating effect is demonstrated for liquid-phase transmission electron microscopy at low electron flux densities using Au nanoparticles as local nanothermometers. The obtained results are in agreement with theoretical considerations. Furthermore, the impact of beam-induced heating on radiolysis chemistry is estimated and the consequences of the effect are discussed.

## Abstract 2

Electron-beam induced heating in the vicinity of a gas bubble in liquid-phase TEM is quantified *in situ*.

## Graphical abstract

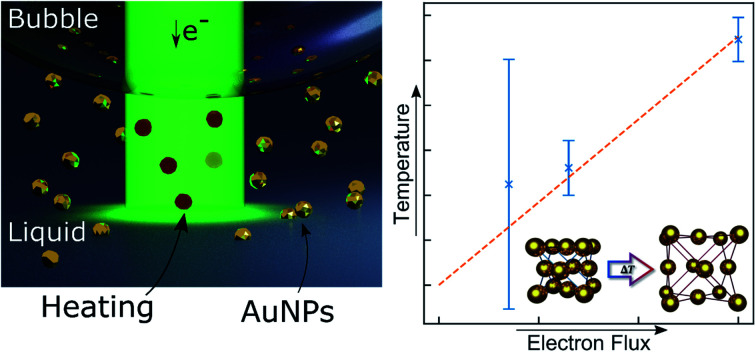

## Introduction

Electron-beam induced effects are crucial for designing experiments and interpreting results obtained by liquid-phase transmission electron microscopy (LPTEM). Most prominently, radiolysis strongly influences solution chemistry governing *e.g.* growth and degradation processes of nanomaterials.^1–4^ In addition, specimens are affected by emerging electric fields^5^ or radiation damage.^6^

One effect, which has been controversially discussed, is beam-induced heating of liquids and of substances dispersed in liquids. Already a small heating effect is expected to be relevant, because not only chemical rate constants, but also electron-beam induced radiolysis sensitively depend on temperature.^7^ Although several attempts to estimate the expected temperature changes have been made, calculations yield partially counterintuitive results and have been not validated experimentally so far. Zheng *et al.*^8^ and Grogan *et al.*^9^ predicted that electron-beam induced heating remains negligible for typical beam conditions. In contrast, Hsieh *et al.* observed an electron-beam induced temperature increase of up to 85 °C at high electron doses by benchmarking nanocrystal formation processes with *ex situ* experiments while using theoretical estimations for modelling the observations.^10^

Recently, Yesibolati *et al.*^11^ applied resistance measurements on electrodes in contact with a liquid phase of glycerol and water to demonstrate that the global heating of a liquid cell during low dose STEM irradiation remains below five Kelvin. Although MEMS-based heating systems allow for high-precision measurements, they only access the global temperature of the TEM holder or at least a liquid volume in the order of several cubic micrometers rather than the local temperature within the irradiated area. The real temperature at the site of observation is not accessible as the heat quickly dissipates to the surrounding liquid by convection and dynamics of thermal exchange.

In order to resolve the problem, there is a strong need for an *in situ* method enabling precise quantification of local temperature changes directly at the location of observation.

Extracting thermal expansion from diffraction patterns of amorphous thin films^12^ or polycrystalline samples^13,14^ has proven to be feasible for thermometry. As these changes are subtle, a strict control of experimental conditions and precise data analysis are required. Particularly parallel-beam electron diffraction (PBED) using gold nanoparticles as temperature sensors demonstrated by Niekiel *et al.*^13^ seems specifically promising for temperature measurements in liquids because it was demonstrated to reach a temperature resolution of ±2.8 K. As gold is a widely investigated material system in LPTEM,^1,4,5,7–9,11,15–25^ the evaluation of electron-beam induced heating on gold nanoparticles is expected to deliver a particularly relevant approach for local temperature evaluation, allowing for improved control and interpretation of *in situ* experiments.

In this communication, we present *in situ* LPTEM experiments, which demonstrate electron-beam induced heating at low electron flux densities. For this purpose, an improved PBED method for measuring temperature changes in LPTEM within the irradiated area is introduced. Its performance is evaluated by comparing temperature profiles from external heating with *in situ* temperature measurements within the irradiated area. We evaluate how the electron beam contributes to local specimen heating. Furthermore, we show that an electron-beam induced temperature increase can be significant even under low-dose conditions when imaging metallic nanostructures in the vicinity of gas bubbles. Finally, we calculate the impact of electron-beam induced heating on the equilibrium concentrations of radiolysis products. These findings are relevant for the vast majority of LPTEM experiments studying, *e.g.*, nucleation and growth of nanostructures, material degradation and corrosion, or even nanoparticle motion.

## Experimental

### Instrumental setup

All TEM experiments were performed using an image and probe corrected FEI TITAN Themis^3^ 80-300 (S)TEM microscope operated at an acceleration voltage of 300 kV. Fine tuning of beam parallelism^13^ is enabled by a three-stage condenser lens system. The set up offers a wide range of illumination diameters with a parallel beam (by the C2–C3 condenser zoom). Selected area electron diffraction (SAED) was performed using an aperture cutting the measurement region down to a diameter of 3.3 μm (the illumination diameter was set to 8–10 μm). The camera length was chosen to capture data until the {222} diffraction ring of gold using a Ceta camera with 4k × 4k pixels.

**Fig. 1:**
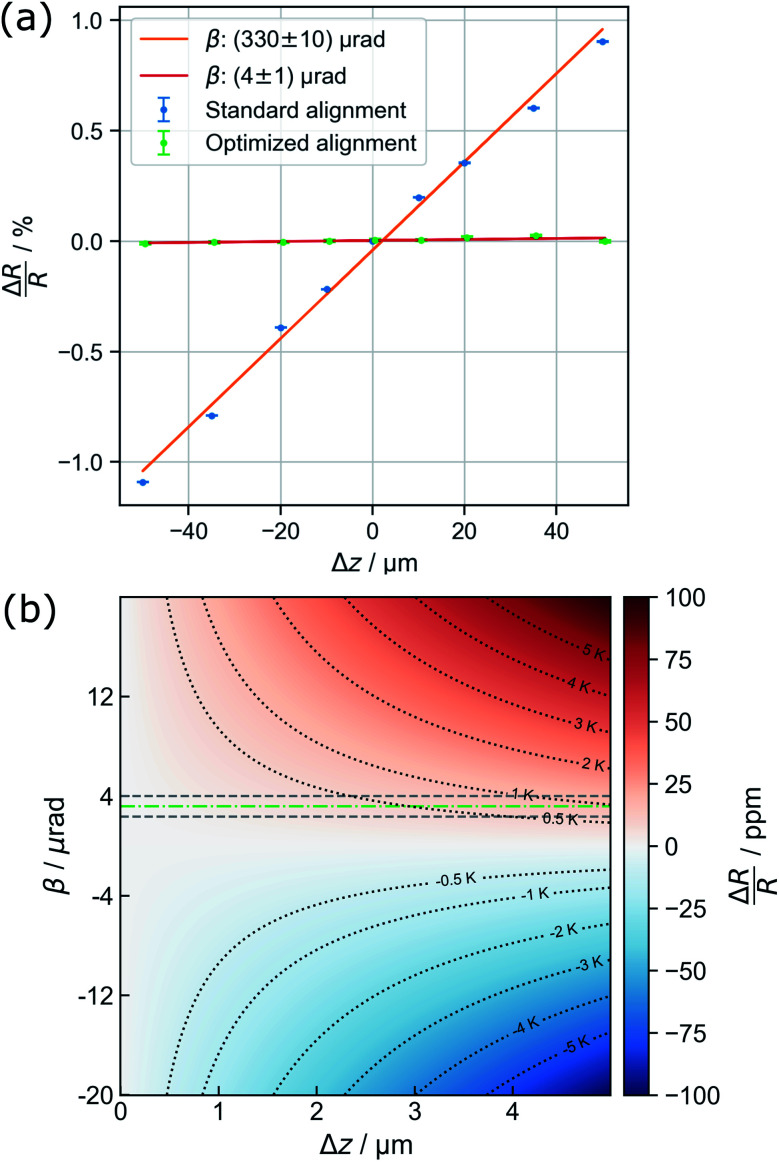
(a) Minimization of the vergence angle by tuning of electron-optical settings, and (b) the residual impact of the systematic error of the *z* displacement on temperature measurements. The dashed dotted line marks the achieved convergence angle shown in (a), whereas the dashed lines show its 2*σ* confidence interval.

Experiments were conducted using the liquid flow holder system Poseidon Select© (Protochips). The utilized liquid cell consists of a top chip providing a viewing area with a size of 50 × 500 μm^2^ and a bottom chip with the same window size equipped with a gold spacer layer with a thickness of 150 nm. The top chip can be heated by a tungsten-based heating coil, which is controlled *via* monitoring its thermal coefficient of resistivity (TCR). Both chips were prepared following the recommended protocols from Protochips by removing the PMMA protection layer in acetone and methanol and blow-drying with N_2_. To mitigate potential charging effects reducing the obtainable resolution during TEM,^26^ the outer membranes of both chips were coated with an amorphous carbon (a-C) layer. The thickness of a-C was determined to be 9.8 nm by evaluating reflectance spectra acquired by microspectroscopy using a system-corrected^27^ generalized transfer matrix method^28^ (see the ESI, Fig. 1†).

Prior to loading, plasma treatment of the inner membrane surfaces was performed for enhancing wettability. A droplet of deionized water was dispensed onto the viewing area before mounting the chips with an orthogonal alignment of the long window sides.

Parallel-beam alignment is extremely important for attributing changes in the radius of the diffraction rings Δ*R* to a change in the specimen temperature. This is because the relative change (Δ*R*/*R*) of any diffraction ring in focus depends on the position of temperature sensors (*i.e.* the gold nanostructures) along the optical axis (*z*-position, Δ*z*) and the beam vergence (*β*, positive for a convergent beam and negative for a divergent beam):^13^1
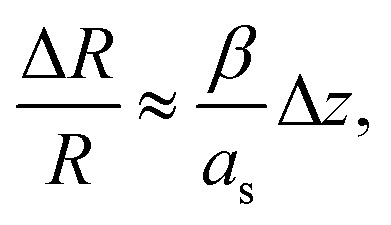
*a*_s_ denotes the radius of the selected area. Consequently, *β* is measured by fitting the relative radius change of the {220} Au diffraction ring as a function of the *z*-position and minimized by carefully adjusting the C2–C3 condenser zoom.^13^ By comparing (factory) standard and optimized parallel-beam alignment, it becomes evident that *β* was reduced significantly and was determined close to zero (4 ± 1 μrad) for the experiments presented here (Fig. 1(a)).

Due to bulging, Δ*z* can range up to a few microns in the utilized setup. Together with the residual non-zero *β*, this results in a detectable radius change that is not related to temperature and, thus, defines a systematic error bound. As illustrated in Fig. 1(b), the maximal error amounts to about ±1 K for the setup used during the presented experiments. To exhaust this margin, a movement of temperature sensors of 5 μm along the optical axis is required.

The electron flux density *ϕ* was adjusted by tuning the monochromator. To prevent possible beam-induced artefacts, the beam was blanked for at least five minutes prior to each temperature measurement (*i.e.* until *t* = 0 in Fig. 3(a) and ESI, Fig. 5a and b†).

**Fig. 2:**
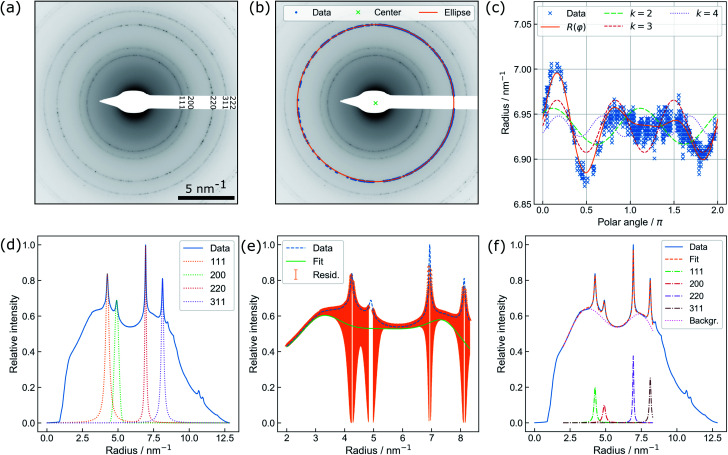
(a) Exemplary diffraction pattern. (b) Illustration of the non-iterative least-squares ellipse fitting to {220} for accurate center finding. (c) Illustration of distortion modelling in polar coordinates to {220} based on eqn (2): Besides the full function, the underlying distortions of *k*^th^ order are drawn. Note that the beam stopper crosses the {220} ring at 1.5*π*. (d) Fit of the Voigt functions to the prominent Bragg peaks and (e) the residual background utilized for creating initial guesses for a combined spectrum modelling as shown in (f).

**Fig. 3:**
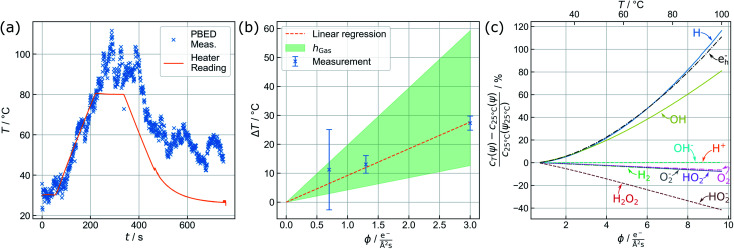
(a) Active heating protocol of a TEM liquid cell (orange) and *in situ* temperature profile acquired from parallel beam electron diffraction by Au nanoparticles (x) in the observation area. Particle-based measurements perfectly reproduce the temperature ramp (until 220 s) but show an offset related to beam heating effects upon subsequent delay and decline. (b) PBED-derived increase of specimen temperature after 5 min of illumination depending on the electron flux density *ϕ*. Error bars mark the maximum deviation of the {220} and {311} data from the mean value. The green area spans the considerable range of temperature variation modelled for a gaseous environment (see text). (c) Beam heating strongly affects the equilibrium concentrations of water radiolysis products and thus the prospected chemical environment. Assuming a reference temperature of 25 °C, the curves give the percental change of relevant species at the corresponding dose rate *ψ* against a conventional radiolysis model neglecting beam-heating effects. The corresponding sample temperature in the observation area is plotted on the top axis.

### Preparation of temperature monitoring samples

As nanothermometers, gold particles have proven to be feasible because of their inert nature in pure aerated water without species complexing Au cations (*e.g.* halogenides),^22^ and their high material contrast. These particles were prepared on the inner surface of the silicon nitride top window by sputter deposition of a thin gold layer (18 nm) followed by solid-state dewetting.^29^ Dewetting was performed at a relatively low temperature of 200 °C by heating the top chip in a furnace for 31 h to conserve the polymer passivation of the electronics. ESI, Fig. 3(a) and (b)† show the STEM images of gold particles in the liquid after the experiments have been conducted. Although the particles were only deposited on the top membrane (a), it is evident that a distinct fraction of particles has been detached from the top membrane (b) during the experiments. Due to bulging, the maximum distance between the membranes (at the center of the viewing area) was determined to 4.2 μm by STEM defocus. This underlines the requirement for parallel beam alignment during thermometry because the particles are clearly distributed inside the entire volume of the liquid cell.

### Temperature calculations

To attribute the change of the extracted relative position of the Bragg diffraction rings to temperature changes, the relative thermal expansion of the Au lattice parameters is modelled by a polynomial of third order.^30^ The coefficient of thermal expansion (CTE) can safely be approximated using bulk values because the mean diameter of particles clearly exceeds 10 nm ^31^ (ESI, Fig. 3(c)†). In the case of external heating, the starting temperature was set to the temperature evaluated using the heating device prior to heating. For experiments without heating, a starting temperature of 20 °C (room temperature inside the lab) was assumed.

### Data evaluation

An automated data analysis algorithm is used for precise determination of the relative change of the lattice constant based on polycrystalline diffraction patterns (Fig. 2(a)). Residual image distortions in the diffraction patterns are corrected up to the fourth order and the radial profile is modelled using a set of Voigt functions. The data analysis routine is based on a workflow presented in our previous study on *in situ* temperature measurements in a vacuum,^13^ but is optimized to operate for data comprising faint diffraction rings and varying amorphous background contributions. In particular, the routine does not rely on precise value guesses for the center position of the direct beam, radii of diffraction rings, and amorphous background distributions. The latter is especially important, as background contributions are prone to change during LPTEM, for example due to bubble formation.^9^

The algorithm is written in Python 3.7 and uses Hyperspy,^32^ NumPy,^33^ Matplotlib,^34^ pandas,^35^ scikit-image^36^ and SciPy.^37^ To extract the radial profile, polar transformation has to be applied with respect to the center of the diffraction pattern. The center position, however, is not directly accessible since the beam stopper (Fig. 2(a)) blocks the direct beam. Therefore, a center guess based on a circular Hough transformation is derived from the diffraction pattern. Polar transformation is performed using PyAbel.^38^

Peak finding^39^ is utilized for pixel extraction of the diffraction rings of interest, combined with intensity-based thresholding. The range of ring radii is constrained by reasonable input variables whereas the center of the respective diffraction ring is determined using non-iterative least-squares ellipse fitting,^40,41^ as illustrated in Fig. 2(b).

Finally, distortions are interpreted as the azimuthal (*φ*) variations of the radius *R* of a diffraction ring which is the product of the undistorted radius *R*_0_ and the sum of circular distortions up to the 4^th^ order. This is performed by least-squares fitting of eqn (2) to the extracted ring data, as demonstrated for the {220} ring in Fig. 2(c).2
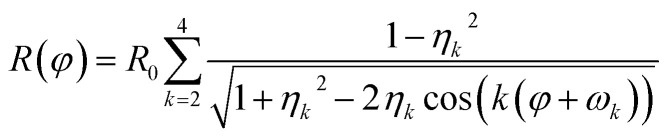
here, *η* describes the amplitude of the distortion of the *k*^th^ order, whose orientation of the major axis is determined by *ω*_*k*_. The intensity of diffraction spots (*i.e.* the pixel grey value) was used as a weighting factor.

Subsequently, the distortion-corrected, radially integrated diffraction pattern is described by a set of Voigt functions to simultaneously model the Bragg peaks and the amorphous background. The data used for fitting exclude the radial range of the diffraction pattern dominated by the beam stopper tip (small radii), and the missing information because of the squared detector symmetry (large radii), respectively. Center finding and distortion correction are performed for each ring individually.

During LPTEM, reference measurements without crystalline particles for modelling the amorphous background are not feasible due to variations in the background caused by specific dynamics during the experiments. In particular, bubble formation, motion of particles and sample drift, as well as radiolytically-induced particle formation or degradation dynamically change the intensity ratio between the Bragg peaks and the amorphous background.

Thus, the least-squares fit of the spectrum relies on the automatic creation of initial guesses and the restriction to physically meaningful parameter boundaries (*i.e.* no negative peak maxima and widths). First, the individual Au diffraction peaks are fitted separately using a Voigt profile (Fig. 2(d)). Initial fit parameters are obtained by using the theoretical lattice constant of gold for calculating the relative peak positions of individual diffraction rings. The radius obtained during distortion fitting is used as a reference. Second, the residual intensity profile is modelled by a set of three Voigt functions. The difference of the residual and original data (orange area) is minimized during the least-squares fit (Fig. 2(e)). Finally, the resulting parameters are used as starting values for a simultaneous fit of the full spectrum (Fig. 2(f)). In the case of a successful fit, the resulting parameters are used as starting values for fitting of the next diffraction pattern. This comprehensive procedure enables precise determination of the Au lattice spacing from diffraction patterns with varying background contributions and is therefore specifically suitable for *in situ* LPTEM experiments.

## Results & discussion

In order to measure temperature *via* PBED, the sample thickness should not be significantly larger than the inelastic mean free path of the sample (about 380 nm in water for the setup used in our experiments^42^) because a poor signal-to-background ratio at thicker samples will limit the precision of the peak fitting. Although this thickness limit can be further extended using elastic filtering (see the ESI, Fig. 2†), it was not possible to obtain a feasible diffraction signal at a filled and bulged cell.

To reduce the thickness of the liquid an ambient gas bubble was intentionally introduced during the loading process of the liquid cell (see the ESI, Fig. 4†). By taking the Si_3_N_4_ membranes and a-C layers into account, the residual (projected) liquid thickness in the region of the gas bubble amounted to 140 nm as measured by the electron energy loss spectroscopy (EELS) log-ratio method.^43^ Intensity variations, however, suggest minor liquid thickness fluctuations during the experiments. Furthermore, particle motion is observed in both real and reciprocal space (see the ESI, Video 1†), emphasizing the presence of the liquid layer. We note that we do not observe any particle degradation during the whole experiment.

### Accessing the local specimen temperature during LPTEM

The capability of measuring changes in temperature *T* over time *t* during LCTEM was validated by applying a temperature profile using the heating capability of the TEM holder upon irradiation with an electron flux density *ϕ* of 5 e^−^Å^−2^ s^−1^. Fig. 3(a) shows the comparison of the resistivity-based reading of the heating device with the evaluated temperature derived from the {220} data.

A temperature plateau at 30 °C was established, before applying a heating ramp of 0.3 K s^−1^ up to 80 °C. It is evident that temperature measurements *via* PBED and device reading are in good agreement during this initial phase of the measurements. To quantify the precision of the method, the sample standard deviation in the plateau at 30 °C (50 frames) is calculated, amounting to ±2.88 K. Despite a significantly increased amorphous background contribution, this is comparable to our previous temperature measurements by PBED in vacuum,^13^ indicating a significant improvement of the evaluation procedure.

Following the heating ramp the temperature was held constant at 80 °C for 120 s. At this point, reading and measurements start to deviate. During this time the temperature reading only documents a negligible (global) temperature overshoot of 0.6 K, whereas the PBED measurements reveal an (local) increase of more than 10 °C. It is furthermore evident that the thermal fluctuation increases significantly, indicating that dynamic processes affect the local temperature.

Finally, the chip heater was switched off while continuously measuring the local temperature until the chip reading settled at 25 °C, which corresponds to the starting temperature measured by the heating device. The corresponding temperature measured *via* PBED decreases with a significant delay, suggesting poor heat dissipation from the analysed Au nanoparticles. Most striking, however, is that the final temperature does not return to the initial value during the time of observation but stabilizes at around 50 °C. Note that we have observed this discrepancy regularly (see the ESI, Fig 5†).

To interpret these deviations, it is crucial to bear a few considerations in mind. The heating coil is neither calibrated individually (for a particular heating chip) nor are the resistivity measurements performed directly on the chip (*e.g. via* four point probe measurements close to the viewing area), as this is standard for *in situ* heating devices in comparable systems operating in a vacuum or gas environment. Nevertheless, the good agreement of both, PBED and resistivity measurements for the first 220 seconds shown in Fig. 3(a) justifies the assumption that both methods work correctly.

The measurements, however, are performed at different positions within the experimental setup. The heating coil surrounds the outer rim of the large silicon top chip and is, thus, millimetres away from the observation window. This underlines the necessity to analyse the temperature as close to the spot of observation as possible, ideally directly within the irradiated area.

The rate difference in cooling compared to heating may be related to a size effect of the nanocrystals themselves. Recent work suggest that nanoparticles heat up faster than they cool down, when their motion can be described by overdamped Langevin equations,^44^ such as Brownian motion or diffusion,^45^ which is typical for nanoparticles enclosed in liquid cells.^8,11,18,22^ Additionally, the gas bubble introduced to enable PBED in the first place causes a significant thermal isolation of the irradiated area, as discussed in the following section.

### Influencing the local temperature by electron irradiation

After demonstrating that PBED is capable of measuring thermal changes during *in situ* LPTEM, we irradiated the sample area without additional external stimuli (*i.e.* heating coil switched off) at different dose rates *ψ* to evaluate a possible heating effect. Irradiation was performed for five minutes at different electron flux densities *ϕ*. Between the measurements, the system was allowed to settle for ten minutes. *ϕ* and *ψ* are related to each other *via* the following equation derived in the ESI†:3
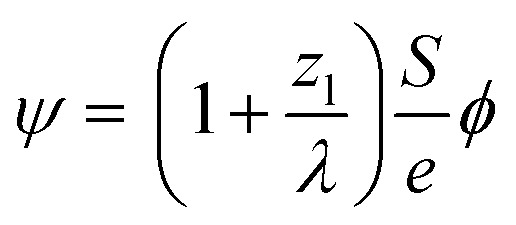
here, *S* denotes the stopping power, *e* the elementary charge, *z*_l_ the liquid thickness and *λ* the inelastic mean free path in water. Eqn (3) is illustrated in the ESI, Fig. 6.†

Fig. 3(b) displays the averaged temperature after 5 min of irradiation derived from the {220} and {311} diffraction rings. The corresponding temperature profiles are presented in the ESI (see ESI, Fig. 7†). To clarify the results, the temperature increase at 1.3 e^−^ Å^−2^ s^−1^ has been re-measured, yielding four instead of two individual measurements for this particular data point. The error bars correspond to the maximum deviation of the measurements from the mean value. At 1.3 e^−^ Å^−2^ s^−1^ and at 3.0 e^−^ Å^−2^ s^−1^, the uncertainties are in good agreement with the precision obtained in the previous section. For 0.7 e^−^ Å^−2^ s^−1^, the error drastically increases, which can be attributed to the small electron flux density itself lowering the obtained signal-to-background ratio.

In contrast to previously published results at similar flux densities and illumination duration,^11^ a direct proportionality between temperature increase and the electron dose rate is visible in Fig. 3(b). Although this trend is predicted by theory, its magnitude is significantly larger than expected. As elucidated before,^8,9,46^ electron beam interaction solely with the liquid layer itself is unlikely to yield a thermal increase to such an extent. Electron-beam heating of nanoparticles and heat exchange with the surrounding, in turn, play a dominant role.^8^

By combining these considerations with dose- and thickness-dependent heating of the residual liquid film,^46^ it is possible to estimate the expected electron-beam induced heating as a function of the dose rate and material parameters for the experimental conditions present in this study (see the ESI† for details):4
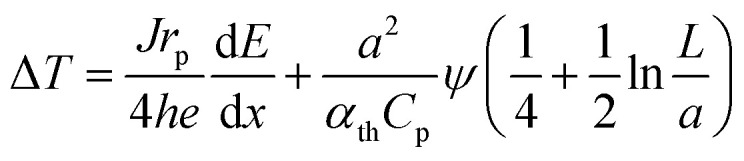
here, *h* is the heat transfer coefficient of the surrounding material. *J* denotes the beam current density, *r*_p_ is the particle radius (see the ESI, Fig. 3(c)†), *α*_th_ and *C*_p_ are the thermal diffusivity (1.4 × 10^−7^ m^2^ s^−1^), and specific heat capacity of water (4.18 J g^−1^ K^−1^), respectively.^46^*L* denotes the liquid pocket side length of 2/√*π* × 50 μm ^46^ and *a* is the beam radius (5 μm). At an acceleration voltage of 300 kV, the energy dissipation d*E*/d*x* amounts to 261 meV nm^−1^ in Au.^8^Eqn (3) was used to convert *ϕ* into *ψ*. Consequently, only *h* remains unknown.

To estimate *h*, a weighted linear regression was performed. The result is plotted in Fig. 3(b) (orange, dashed line), yielding a heat transfer coefficient of 4.37 W m^−2^ K^−1^ and a corresponding slope of 9.22 K Å^2^ s/e^−^. For liquid water, *h* amounts to ∼10^3^ W m^−2^ K^−1^.^8^ Whilst heat dissipates effectively in the bulk liquid, a gaseous phase dominating the heat transport dramatically changes the situation, so that *h* is expected to range between 2 and 10 W m^−2^ K^−1^.^47^ The green shaded area in Fig. 3(b) indicates this range, suggesting that the reduced heat transfer observed is more likely attributed to thermal isolation provided by the nearby gas bubble.

It must be considered that the above calculations do not account for potential heat transfer across the membranes which could lower the heating effect. On the other hand, gold nanostructures may significantly increase the energy absorbed by the sample and thus the dose rate (and consequently the expected heating) locally due to secondary electron emission.^24^

The observed beam heating effect explains well why the measured temperature profile in Fig. 3(a) does not settle at room temperature after switching off the external heating, but fluctuates between 50 and 60 °C. According to Fig. 3(b) a sample temperature in this range is expected to result from beam induced heating at the used electron flux density *ϕ* of 5 e^−^ Å^−2^ s^−1^. Experiments at larger flux densities up to 11.2 e^−^ Å^−2^ s^−1^ were performed as well and follow the same trend. However, a solid evaluation and interpretation of these measurements remained difficult, due to strong fluctuations in the diffraction signals that we assign to increased turbulences in the liquid and bubble dynamics.

These findings open up a possibility for more precise *in situ* parameter control during LPTEM. Introducing a gas bubble, thus, can not only be used to enable higher resolution, but may also serve as a tool for locally controlling the temperature even without a dedicated TEM holder or a heating system.

### Impact of electron-beam induced heating on radiochemistry

Whilst electron-beam induced heating can change the local temperature by adjusting the electron flux density, the influence on radiochemistry inside the aqueous phase has to be discussed carefully when designing experiments. To quantify this effect, a simplified radiolysis model introduced by Ambrožič *et al.*^7^ considering temperature-dependent reaction constants and generation values (*G*-values) was implemented in Python using NumPy and SciPy. This reaction set assumes that electron beam irradiation of aerated H_2_O does not create O^−^, O_3_, O_3_^−^, or HO_3_ in a significant amount. Therefore, only a subset of emerging species compared to that reported by Schneider *et al.*^4^ (H, H^+^, H_2_, H_2_O_2_, HO_2_, HO_2_^−^, O_2_^−^, O_2_, OH, OH^−^, and solvated electrons (e^−^_h_)) is regarded.

By controlling the specimen temperature *via* varying the electron flux density, two driving forces change the equilibrium concentrations *c* of the radiolysis products. On the one hand, the local temperature change alters both, *G*-values, and rate constants. On the other hand, the equilibrium concentrations of the radiolysis products directly depend on the dose rate, which is proportional to *ϕ* (eqn (3)). On the other hand, the local temperature change additionally alters both, *G*-values and rate constants. To describe the influence of electron-beam heating on the concentrations of the respective species, the difference between the dose rate- and temperature-dependent concentration *c*_T_(*ψ*) and a solely *ψ*-dependent concentration *c*_25°C_(*ψ*) must be investigated.

By considering the proportionality between the temperature increase and electron flux density measured above, the dependency of the equilibrium concentrations on the electron flux density was calculated between 20 °C and 100 °C including the heating effect. The results are shown in Fig. 3(c). To simplify the comparison between the species, all values are furthermore normalized to the respective equilibrium concentrations obtained when the electron beam was used to heat the system up to 25 °C *c*_25°C_(*ψ*_25°C_). The absolute values of the steady state concentrations including the temperature effect are plotted in the ESI, Fig. 8.†

It is clearly visible that the additional heating influences the H, e^−^_h_ and OH concentration the strongest, whose concentrations increase exponentially with temperature. On the other hand, the equilibrium concentrations of H_2_O_2_, and HO_2_^−^ are slightly reduced, which is not surprising due to their strong coupling to H and OH within the reaction set. This is expected to cause significant changes in investigations of growth and degradation processes observed by LPTEM. All other species do not show a significant heating-related concentration change. Particularly H^+^ and OH^−^ remain unaffected, indicating that beam-induced heating does not alter the pH value, thus, ensuring a purely dose-dependent acidity control.

These considerations must not be confused with concentration changes caused by external heating at a constant dose rate. As demonstrated previously,^7^ this can still lead to a significant change in the equilibrium concentrations. Yet, they reveal for which species the *T*-induced concentration variation is negligible compared to the mandatory changes in the dose rate.

As the system is open, the pressure within the cell should yield roughly one bar. Interactions between the gaseous and liquid phase in the regarded system are thus considered to be minor^7^ and are therefore neglected.

Previous work indicates that diffusion in the vicinity of a gas bubble is strongly hindered.^22^ Consequently, diffusion of the species out of the irradiated area is not taken into account.

## Discussion

The achievable thermal resolution is a parameter of the acquisition time. Consequently, the trade-off between thermal and temporal accuracy requires careful adjustment of the acquisition time. As discussed previously,^13^ the resolution furthermore depends on the number of particles within the selected area. Due to the very nature of SAED, the selected area aperture is defining the spatial resolution of the method. In our case, this is significantly smaller than the illuminated area during PBED.

It is crucial to keep in mind that the measured temperature presented in this work does not directly relate to the liquid temperature itself but reflects the temperature of the embedded Au nanoparticles. Although solvent temperature would be theoretically accessible by electron scattering, as well,^48^ the vast majority of LPTEM experiments aims at analyzing processes of nanostructures enclosed in the liquid rather than studying the liquid itself. Thus, the temperature of the nanocrystals is particularly interesting for practical considerations. Nevertheless, due to the high thermal conductivity of both, water, and gold a thermal equilibrium between particles and liquid can be safely assumed.

We emphasize that these results are only spotlights in the high dimensional parameter landscape of LPTEM experiments and should be treated with care when transferring the findings to other conditions. Besides electron flux density and the acceleration voltage, the thickness of the liquid, and its flow rate as well as composition are crucial parameters when accessing electron-beam induced effects. As the demonstrated heating effect is most likely driven by interaction of the electron beam with the nanostructures (here gold nanoparticles), the situation is expected to change significantly when illuminating low-*Z* materials. Also, when performing STEM, the situation is expected to be remarkably different from that of TEM, because the beam scans over the area of interest instead of illuminating the sample homogeneously and continuously. This may alter the dynamics of energy transfer and heat dissipation within the sample. Furthermore, the architecture of the liquid cell itself could significantly affect heat conductivity. Cell architectures and experimental set ups allowing for sufficiently small liquid layers enabling diffraction without a dominating gas bubble (*e.g. via* bulge control^49^) are expected to yield significantly reduced electron-beam induced heating.

## Conclusions

In summary, we demonstrate local temperature measurements within the irradiated sample area during *in situ* LPTEM with a precision of ±2.88 K using parallel beam electron diffraction (PBED) with gold nanoparticles and an improved workflow and data evaluation scheme. By applying this technique, we show that electron-beam irradiation induces significant heating during LPTEM even at low electron fluxes when operating in the vicinity of a gas bubble. Finally, the impact of electron-beam induced heating on radiolysis in liquid H_2_O by varying the local dose rate is estimated, allowing for more precise prediction of radiolytically-induced chemistry in LPTEM.

## Author contributions

B. Fritsch, A. Hutzler and M. Wu designed and performed the experiments. Data curation, *i.e.* the development of the diffraction data analysis routine was performed by B. Fritsch, with crucial suggestions from A. Hutzler, M. Wu, M. P. M. Jank, M. März, and E. Spiecker. A. Hutzler performed the a-C layer thickness determination, including data curation and analysis. The radiolysis simulation tool was written by B. Fritsch and S. Khadivianazar. Data interpretation was performed by B. Fritsch, A. Hutzler, M. Wu, M. P. M Jank, and E. Spiecker. L. Vogl and M. Wu prepared the Au nanoparticles. Theoretical calculations and adaptions to radiolysis shown in Fig. 3(b) and (c) were performed by B. Fritsch and A. Hutzler. B. Fritsch wrote the original draft of the manuscript, which was critically reviewed by all authors. M. März, M. P. M. Jank, A. Hutzler and E. Spiecker supervised the project.

## Conflicts of interest

There are no conflicts to declare.

## Supplementary Material

NA-003-D0NA01027H-s001

NA-003-D0NA01027H-s002
