# Supplementary material for: Accessing local electron-beam induced temperature changes during in situ liquid-phase transmission electron microscopy
Source: Nanoscale Adv. 2021 Feb 19;3(9):2466–74. doi: 10.1039/d0na01027h (PMC9419575; doi:10.1039/d0na01027h)
Supplement: NA-003-D0NA01027H-s001 [file NA-003-D0NA01027H-s001.pdf]

Electronic Supplementary Material (ESI) for Nanoscale Advances.
This journal is © The Royal Society of Chemistry 2021
Please do not adjust margins

Electronic Supplementary Information

Accessing local electron-beam induced temperature changes during *in situ* liquid-phase transmission electron microscopy

Birk Fritsch,§[a] Andreas Hutzler,§[a*] Mingjian Wu,[b] Saba Khadivianazar,[a] Lilian Vogl,[b] Michael P.M. Jank,[c] Martin März,[a,c] and Erdmann Spiecker,[b]

Supplementary Videos

Supplementary Video 1: *In situ* diffraction video captured with twelve frames per second and 1k x 1k pixel resolution illustrating particle motion by flickering diffraction spots.

Thickness determination of the deposited carbon layer

For determination of the layer thickness of the amorphous carbon (a-C) film deposited on top of the liquid cell microchip, a similar a-C film was deposited onto a custom-made optical substrate using identical deposition parameters. It consists of a SiO_2 layer with a known thickness of 1.5 µm, which was grown via wet thermal oxidation on a single-crystalline silicon substrate. The film thickness was obtained by fitting a system corrected[27] physical model[28] to reflectance spectra measured via microspectroscopy by utilizing the layer thickness as the only fit parameter. Refractive index spectra of a-C and Si were taken from Arakawa[S1] and Green[S2] whereas the refractive index of silicon oxide was measured via spectroscopic ellipsometry. This procedure yielded a layer thickness of a-C of 9.8 nm. The results are shown in **Supplementary Figure 1**.

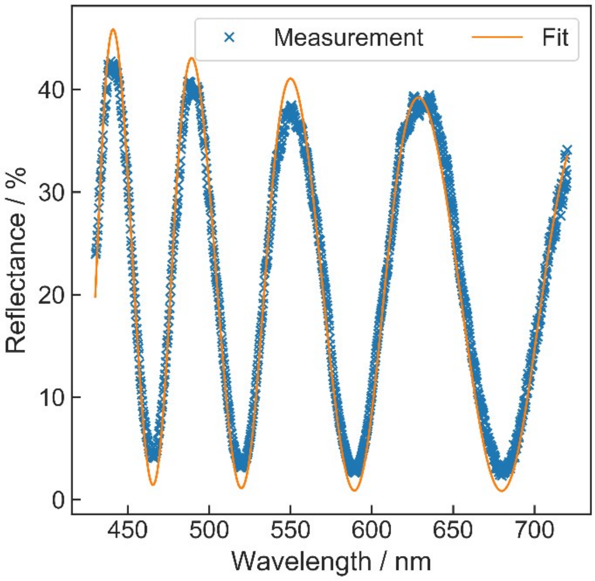

Supplementary Figure 1: Reflectance microspectroscopy of an amorphous carbon film on a SiO_2 layer (1.5 µm) on a silicon substrate and corresponding theoretical calculation of the reflectance spectrum revealing a layer thickness of about 9.8 nm.

Contrast enhancement by energy filtering

As shown in **Supplementary Figure 2**, elastic filtering can be used to enhance the contrast of diffraction patterns during LPTEM. Elastic filtering was performed using a Gatan Image Filter (GIF) with a 10 eV energy slit around the zero loss peak.

Elastically filtered electron diffraction is especially suitable for investigation into specimen inside thick liquid pockets by enhancing the signal-to-background ratio (SBR, Au diffraction vs. diffuse scattering by water and membrane material). By comparing **Supplementary Figures 2(a)** and **(b)** it is clearly visible that the diffraction rings are more pronounced compared to unfiltered diffraction measurements.

When using the microscope setup described in the experimental section of the manuscript, elastic filtering comes at the cost of additional image distortion, a limited pixel resolution of 2k x 2k, and of shadowing a significant part of the pattern by the beam stopper due to the limited camera length available. As for layer thicknesses below the inelastic mean free path of water, the SBR of conventional diffraction is still sufficient for data analysis. Thus, we decided not to perform

a. Electron Devices (LEB), Department of Electrical, Electronic and Communication Engineering, Friedrich-Alexander University Erlangen-Nürnberg (FAU), Cauerstraße 6, 91058 Erlangen, Germany.
b. Institute of Micro- and Nanostructure Research (IMN) & Center for Nanoanalysis and Electron Microscopy (CENEM), Interdisciplinary Center for Nanostructured Films (IZNF), Department of Materials Science and Engineering, Friedrich-Alexander University Erlangen-Nürnberg (FAU), Cauerstraße 3, 91058 Erlangen, Germany
c. Fraunhofer Institute for Integrated Systems and Device Technology IISB, Schottkystraße 10, 91058 Erlangen, Germany.
§ These authors contributed equally
*E-mail: andreas.hutzler@fau.deandreas.hutzler@fau.de

Please do not adjust margins

Please do not adjust margins

elastic filtering for the data shown in the main manuscript. Nevertheless, an exemplary measurement using elastic filtering is shown below in **Supplementary Figure 5(b)**.

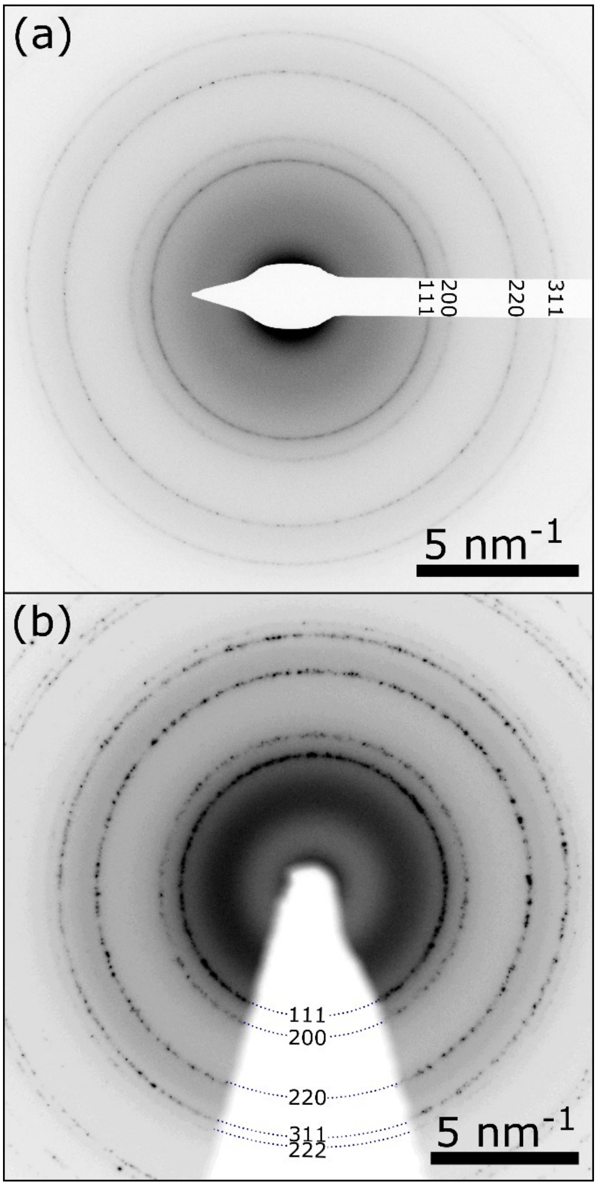

Supplementary Figure 2: Contrast-enhanced polycrystalline diffraction patterns of Au nanoparticles in liquid **(a)** without and **(b)** with elastic filtering.

Real space images

In **Supplementary Figure 3(a)** and **(b)** bright field STEM images of the liquid cell are depicted. In **(a)** the top and in **(b)** the bottom membrane is in focus. It is noteworthy that Au particles were only dewetted on the top membrane, meaning that the particles reached the bottom membrane via diffusion. In **Supplementary Figure 3(c)** the particle radius distribution of spheres with equivalent cross section of 379 particles in **Supplementary Figure 3(a)** is plotted. A Gaussian fit reveals a radius maximum of 37.2 nm. The image analysis yielding the shown particle distribution was performed using FIJI[S3].

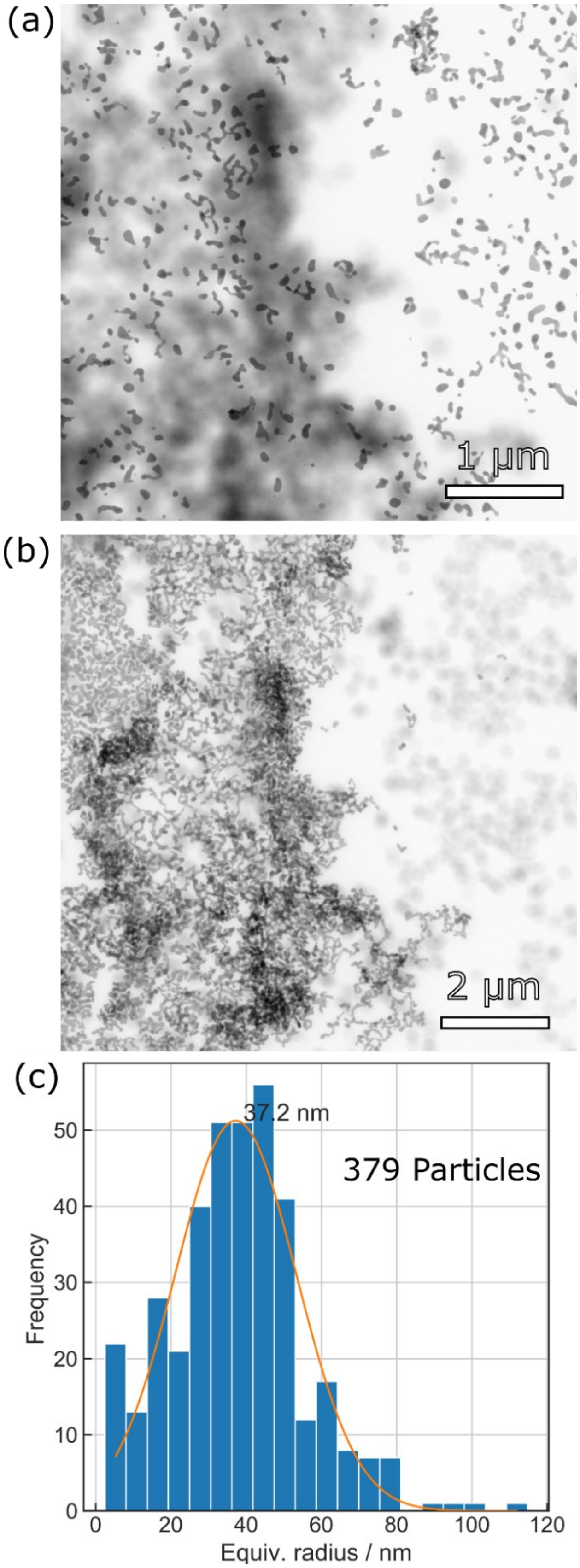

Supplementary Figure 3: Bright field STEM images of **(a)** the top and **(b)** the bottom membrane after conducting the experiments. **(c)** Histogram and Gaussian fit (orange curve) of the particle distribution shown in **(a)**.

This journal is © The Royal Society of Chemistry 20xx

Please do not adjust margins

Please do not adjust margins

Supplementary Figure 4 shows a micrograph of the micron-sized gas bubble encapsulated within the liquid cell obtained by bright-field TEM. At the corners, the thick liquid layer is visible as dark areas. Flux density-dependent measurements were performed in the centre of the bubble.

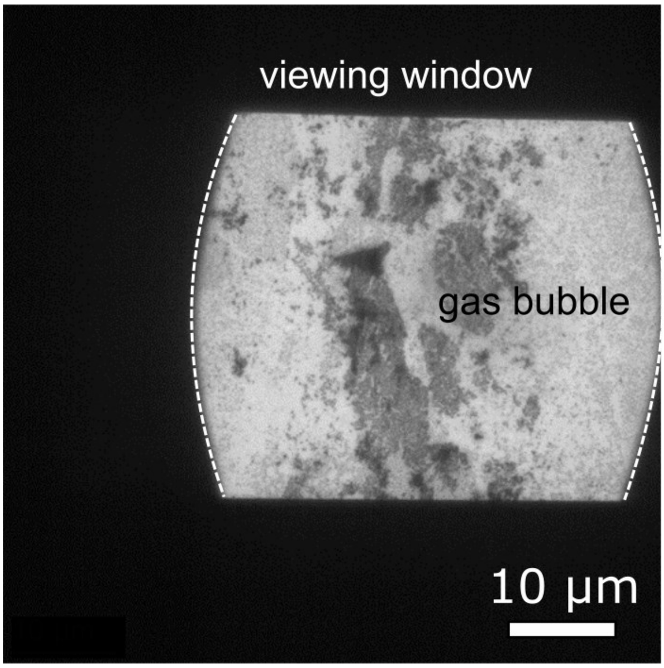

Supplementary Figure 4: Low magnification bright-field TEM micrograph of the gas bubble within the viewing area of the used liquid cell.

Additional verification experiments to measure temperature changes during LPTEM via PBED

In **Supplementary Figure 5**, *in situ* temperature profile measurements performed on a different chip than the data presented in the main manuscript are depicted. **Supplementary Figure 5(a)** shows a profile up to 70°C acquired with 2k x 2k pixel resolution, whilst **Supplementary Figure 5(b)** shows an elastically filtered measurement of a ramp up to 100°C. Here, the heating was not switched off after the plateau but the chip was set to cool down slowly. It is obvious, that both PBED measurements shown here do not settle at room temperature as in the case discussed in the main manuscript. It is furthermore evident that, for the present conditions, elastic filtering does not significantly alter the achievable temperature resolution. Nevertheless, elastic filtering is expected to enhance the signal-to-noise ratio and temperature resolution in LPTEM experiments conducted at thicker liquid films.

In **Supplementary Figure 5(b)** a failure of the used heating coil is documented, which is probably related to harsh thermal treatment of the chip during the dewetting process. It is clearly visible that the measured local temperature further decreases as expected, further emphasizing the power of local temperature measurement by PBED without relying on an external electronic readout.

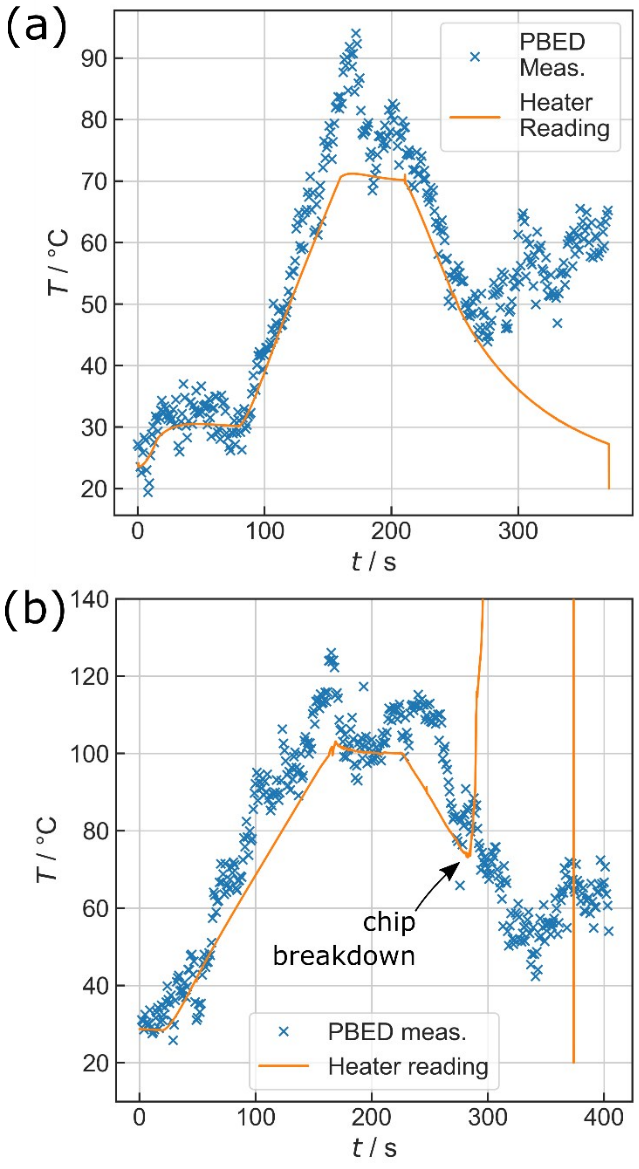

Supplementary Figure 5: Additional temperature measurements using PBED **(a)** without and **(b)** with energy filtering.

Derivation of a dependency between electron flux density and dose rate

The dose rate ψ is defined as the amount of power absorbed by matter. This is fundamentally different from the electron flux density ϕ, which is measuring the amount of electrons *n* per time *t* and unit area A_ϕ.

$$\phi = -n\frac{-e}{A_\phi t} = n\frac{e}{A_\phi t} \quad (1)$$

With –*e* as electron charge and the negative sign to account for the flux direction of negative charges.

Please do not adjust margins

Please do not adjust margins

As derived by N. Schneider[4], ψ is a function of the stopping power S in SI units, the (averaged) distance the electrons travel through the liquid (track length) d, the irradiated volume V_{beam}, and the amount of electrons penetrating the sample per unit time J:

$$\psi = \frac{Sd}{V_{beam}}J \tag{2}$$

J is defined as the amounts of electrons n^* travelling through A_{beam} during a time period t. This is easily convertible to electrons per area A_ϕ.

$$\frac{n^*}{A_{beam}} = \frac{n}{A_\phi} \Rightarrow n^* = n\frac{A_{beam}}{A_\phi} \tag{3}$$

This allows for calculating J as a function of ϕ:

$$J = \frac{n^*}{t} = \frac{nA_{beam}e}{tA_\phi e} = \phi\frac{A_{beam}}{e} \tag{4}$$

When assuming a beam with cylindrical shape inciding normal to the sample (parallel beam), V_{beam} equals the product of a circular base area A_{beam} and the liquid thickness z_l.

$$\psi = \frac{Sd}{A_{beam}z_l}J \tag{5}$$

d is defined by the scattering events in liquid, which is reflected by the (inelastic) mean free path λ. If z_l does not exceed a few microns, this can be described by first order approximation[4]:

$$d = z_l\left(1 + \frac{z_l}{\lambda}\right) \tag{6}$$

Inserting (6) in (5) yields:

$$\psi = \left(1 + \frac{z_l}{\lambda}\right)\frac{SJ}{A_{beam}} \tag{7}$$

With respect to (4), this can be used to convert ϕ into ψ:

$$\psi = \left(1 + \frac{z_l}{\lambda}\right)\frac{S}{e}\phi \tag{8}$$

For an acceleration voltage of 300 kV, S amounts to 2.36 MeV(cm)²/g [S4], and λ is measured to be about 380 nm[42]. The resulting dose rates as a function of ϕ are plotted in **Supplementary Figure 6**. For liquid layers that are significantly thinner than λ, Eq. (8) can be simplified to:

$$\psi = \frac{S}{e}\phi \tag{9}$$

This is in agreement with Alloyeau *et al.*[19]. In **Supplementary Figure 6(a)** it is, however, evident that even for liquid layers with a thickness of tenths of nanometres this simplification introduces small errors, which can be easily avoided by using **Eqation (8)** instead.

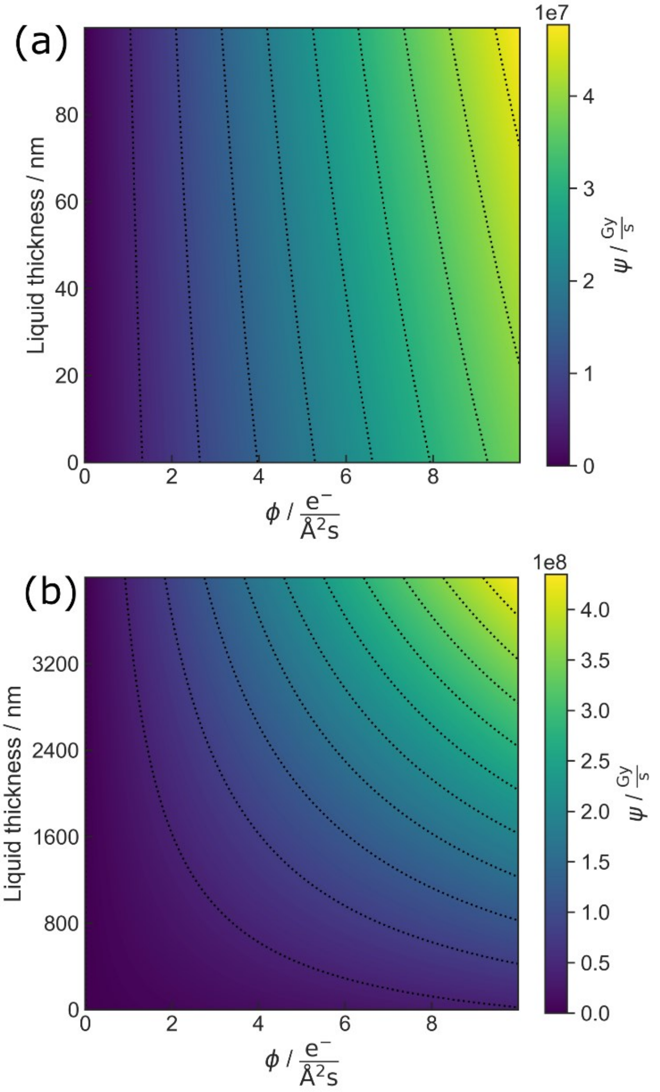

Supplementary Figure 6: Dose rates as function of liquid thickness and electron flux for **(a)** thin and **(b)** thick layer thicknesses. The dotted lines are level lines to illustrate the thickness-dependency of ψ.

Electron-beam induced heating – *in situ* profiles

This journal is © The Royal Society of Chemistry 20xx

Please do not adjust margins

In **Supplementary Figure 7**, the *in situ* temperature profiles of constant illumination for five minutes at varying dose rates without external heating are shown. To ensure an ambient starting temperature, the beam was blanked for at least 10 min prior to every measurement. In lieu of benchmarking the relative radius change against a held set point temperature, the first 30 s of every measurement was set to 20 °C.

Estimation of temperature in the vicinity of a gas bubble in LPTEM

After Zheng *et al.*[8] the electron-beam induced heating ΔT of a particle in a medium is given by

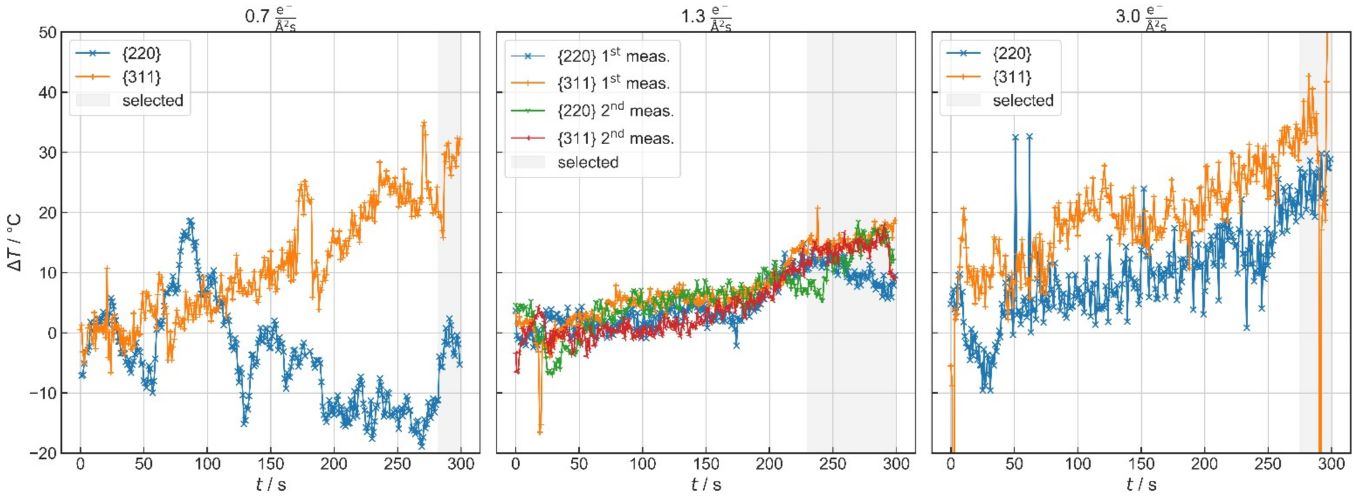

Supplementary Figure 7: Temperature measurements at varying dose rates during five minutes of constant illumination. The data within the shaded region was averaged to extract the trend shown in **Figure 3(b)** of the main manuscript.

At the end of each measurement, a time window (shaded area) was chosen to calculate an averaged final temperature. The plateau was determined in order to try to minimize the trade-off between statistical fluctuation and possible continuation of the electron-beam induced heating. These averaged values are the basis for the data presented in **Figure 3(b)** in the main manuscript.

The data obtained at 0.7 $e^-/(\text{Å}^2 s)$ show drastic fluctuations and deviating trends, which may be attributed to the low dose rate used during data acquisition. This is believed to significantly reduce the achievable temporal resolution. In fact, a stable analysis of the {220} diffraction signal was only achieved by applying a floating average over two frames (the similar procedure was used for the second measurement at 1.3 $e^-/(\text{Å}^2 s)$).

At higher electron flux densities (1.3 and 3.0 $e^-/(\text{Å}^2 s)$), the trends remain consistent between the measurements, which in turn, underline the evidence of observed significant heating.

During the last data acquired (3.0 $e^-/(\text{Å}^2 s)$), the statistical fluctuation of the data is significantly enhanced. This is accompanied with a significant reduction of the relative Bragg peak intensity compared to the amorphous background contribution. This is most likely related to an increase in the dynamics of both, liquid and particle motion, which, in turn, result in flushing the particles out of the observed area. Thermal gradients caused by local heating could trigger these additional dynamics. It is noteworthy that the {311} signal is affected stronger than {220}. This may be related to the clipping of the radii chosen for observation discussed in the main manuscript, which results in a small intensity cut-off of the {311}-Voigt peak at its right flank (see **Fig. 2(f)** for illustration).

$$\Delta T = \frac{J_e\, dE}{4hdX} + \Delta T_{Medium} \qquad (10)$$

Where h is the heat transfer coefficient of the surrounding material in $\frac{W}{m^2K}$. For liquid water, h amounts to $\sim 10^3 \frac{W}{m^2K}$[8]. In air without convection or flow, h ranges between 2 – 10 $\frac{W}{m^2K}$[45]. J_e is the electron current in $\frac{electrons}{m^2 s}$ and is derived from the beam current density J in $\frac{A}{m^2}$ and the elementary charge *e:*

$$J_e = \frac{J}{e} \qquad (11)$$

For a single electron in Au, the total energy dissipation by the electron beam dE/dX is given by the product of the energy dissipation per nm and the particle radius r_p. At 300 kV acceleration voltage, the following relation holds[8]:

$$\frac{dE}{dX} = \frac{dE}{dx} \cdot r_p = 0.261 \frac{eV}{nm} \cdot r_p \qquad (12)$$

r_p is assumed as mean particle radius (see **Suppl. Fig. 3(c)**). T_{Medium} was calculated after N. Schneider[46]:

Please do not adjust margins

$$\Delta T_{Medium} = \frac{a^2}{\alpha_{th} C_p} \psi \left(\frac{1}{4} + \frac{1}{2} \ln \frac{L}{a} \right) \quad (13)$$

α_{th} and C_p are the thermal diffusivity ($1.4 \cdot 10^{-7}$ m²/s), and specific heat capacity of water (4.18 J/(g K)), respectively, and L is the liquid pocket side length of $\frac{2}{\sqrt{\pi}} \cdot 50$ µm[46]. a is the beam radius (5 µm). In sum, the heating effect was estimated by:

$$\Delta T = \frac{J r_p \, dE}{4hedx} + \frac{a^2}{\alpha_{th} C_p} \psi \left(\frac{1}{4} + \frac{1}{2} \ln \frac{L}{a} \right) \quad (14)$$

T-dependent radiolysis simulations

A Python-based adaption of the reaction set developed by Ambrožič *et al.*[7] was used for simulation. We kindly ask the interested reader to refer to the original work for further information on the used reactions, kinetic constants, and G-values. **Supplementary Figure 8** shows the absolute values of the equilibrium concentrations including a considered heating effect. The simulations were conducted until 10^3 s**.**

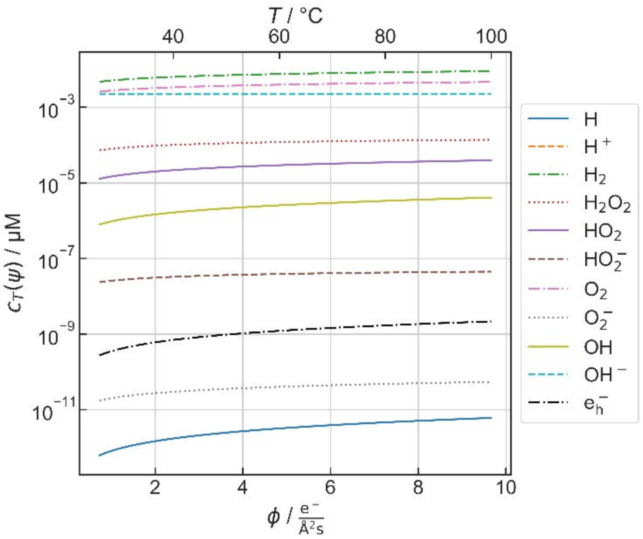

Supplementary Figure 8: Absolute steady state values for radiolysis products of the utilized reaction set.

Supplementary References

S1 E. T. Arakawa, M. W. Williams and T. Inagaki, Optical properties of arc-evaporated carbon films between 0.6 and 3.8 eV, *Journal of Applied Physics*, 1977, **48**, 3176.

S2 M. A. Green, Self-consistent optical parameters of intrinsic silicon at 300K including temperature coefficients, *Solar Energy Materials and Solar Cells*, 2008, **92**, 1305.

S3 J. Schindelin, I. Arganda-Carreras, E. Frise, V. Kaynig, M. Longair, T. Pietzsch, S. Preibisch, C. Rueden, S. Saalfeld, B. Schmid, J.-Y. Tinevez, D. J. White, V. Hartenstein, K. Eliceiri, P. Tomancak and A. Cardona, Fiji: an open-source platform for biological-image analysis, *Nature Methods*, 2012, **9**, 676.

S4 S. Seltzer, *Stopping-Powers and Range Tables for Electrons, Protons, and Helium Ions, NIST Standard Reference Database 124*, 1993, https://physics.nist.gov/PhysRefData/Star/Text/ESTAR.html, (accessed 12 October 2020).

This journal is © The Royal Society of Chemistry 20xx
